# Perioperative Exposure Keratopathy and Corneal Abrasion in an Individual With Eyelash Extensions

**DOI:** 10.7759/cureus.72061

**Published:** 2024-10-21

**Authors:** Elliott Cope, Jade Radnor, Eliza Beasley

**Affiliations:** 1 Emergency, Royal Melbourne Hospital, Melbourne, AUS; 2 Anaesthesia, Northeast Health Wangaratta, Wangaratta, AUS

**Keywords:** blepharitis, corneal injury, cosmetics, eyelash extensions, perioperative

## Abstract

Eyelash extensions are a common cosmetic procedure. However, they are not without risk. In the perioperative context, this may involve a risk of exposure to keratopathy due to corneal surface vulnerability, physical irritation, and/or inadequate eyelid closure. This case documents a patient with dry eye disease likely secondary to their eyelash extensions who subsequently suffered a corneal abrasion with associated morbidity during a routine anesthetic. Furthermore, this case highlights both the importance of preoperative counseling regarding the removal of eyelash extensions in the elective setting and the need for further strategies to mitigate the risk of corneal injuries in these patients.

## Introduction

Eyelash extensions, the cosmetic procedure of attaching semi-permanent false lashes to individual lashes, are increasing in popularity with the global industry expecting a compound annual growth rate of 7.5% during the period of 2024-2032 [1]. This growth is estimated to increase the value of the eyelash extension market from 1.3 billion USD to 20 billion USD by the year 2032 [1]. As such, it is reasonable to expect the rate of patients with eyelash extensions seen in clinical practice to increase. Eyelash extensions are not without medical risks. The most commonly described is blepharitis [2]. This condition is associated with meibomian gland dysfunction and subsequent evaporative tear disorder which can lead to dry eye disease and corneal surface vulnerability [3]. Furthermore, eyelash extensions can also cause lagophthalmos [2] (inadequate closure of the eye) which can lead to dry eye disease, corneal abrasions, persistent epithelial defects, ulceration, microbial keratitis, and corneal scarring [4]. These risks are important within the context of perioperative medicine as they ultimately contribute to corneal vulnerability, which when combined with lagophthalmos increases the risk of exposure to keratopathy and the development of corneal injuries during general anesthesia [5]. This case study describes an individual with eyelash extensions, concurrent blepharitis, and dry eye disease who suffered from exposure keratopathy and received a corneal abrasion while undergoing elective obstetric surgery.

## Case presentation

**Figure 1:**
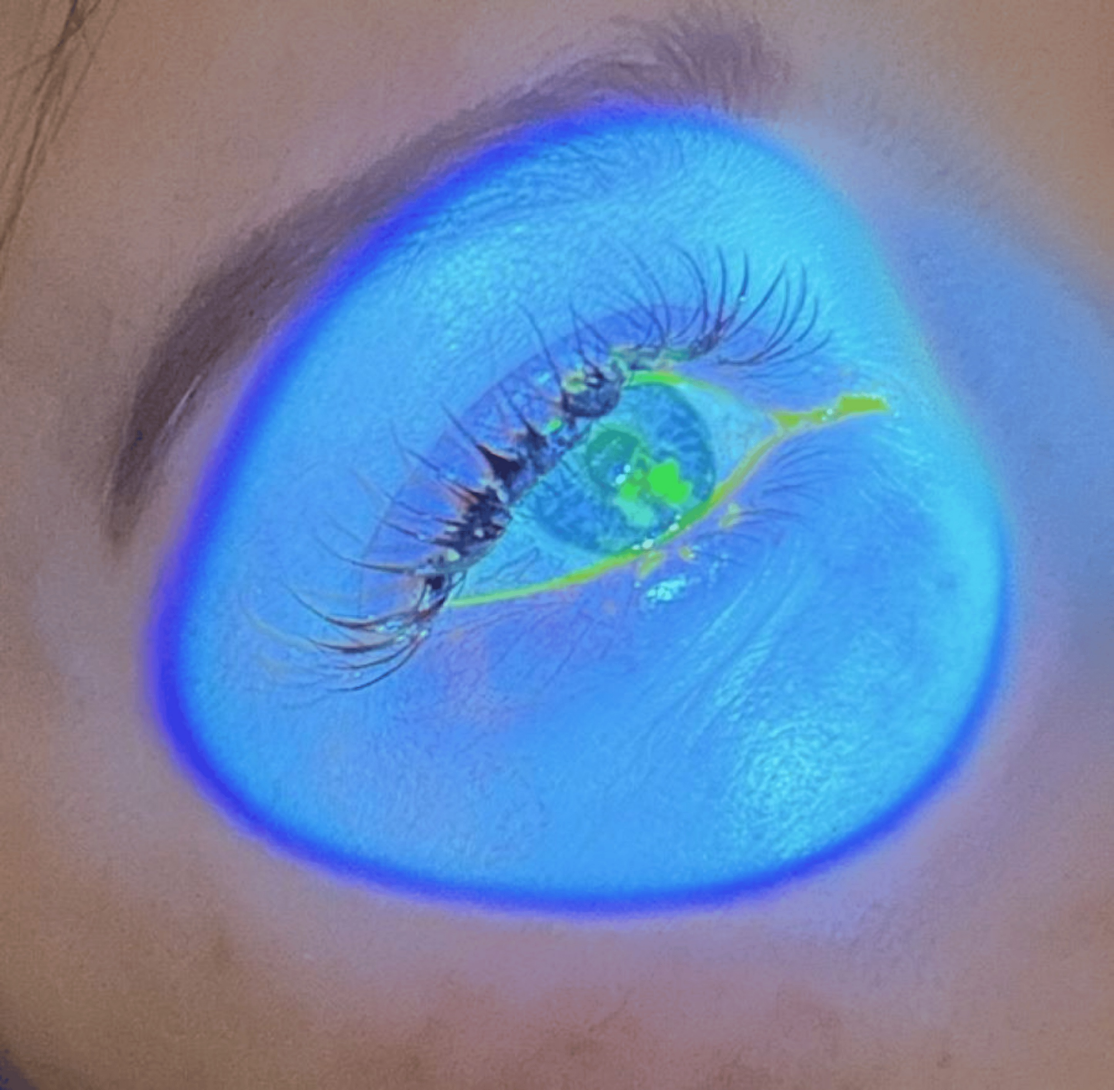
Perioperative corneal abrasion observed under cobalt blue light with fluorescence dye in situ.

A 38-year-old female was scheduled for an elective cesarean section. On history, she had documented bilateral blepharitis with a large chalazion on the right upper eyelid which was awaiting ophthalmological review. The patient indicated that she had been suffering from blepharitis and dry eye symptoms since acquiring eyelash extensions 5 years prior. She was currently using regular preservative-free hydrating eye drops but had not implemented an eyelid hygiene regime. She had no further medical history, her antenatal history was unremarkable, and she was not taking any regular medications. The patient was managed with a general anesthetic and intubated for the procedure due to an inadequate sensory block from neuraxial anesthesia. In the setting of eyelash extensions, Eyepro® covers were unable to completely close the patient’s eyes, as such the patient’s eyes were held close by folded gauze soaked in saline and taped with Transpore™. An ocular lubricant was not utilized as they are not routinely used in the anesthetics department of our healthcare facility. Care was taken during the surgery to ensure no foreign objects came in contact with the covered eyes. The surgery and general anesthetic proceeded uneventfully. During the immediate postoperative period, the patient complained of discomfort in her right eye. Examination of the right eye revealed normal visual acuity (6/6), normal intraocular pressure (16 mmHg), and signs consistent with blepharitis and dry eyes (lid margin hyperemia, telangiectasia, meibomian gland capping, corneal punctate epithelial erosions, and a tear film break-up time <5 s). The patient also had a superficial epithelial corneal defect 0.5 cm × 0.5 cm at the 6 o’clock position adjacent to the limbus (Figure 1).

This was thought to be the primary cause of her complaint. The corneal epithelial defect was treated with regular oral analgesia, a limited debridement, antibiotic and hydration eye drops, and optometry follow-up. By day 7 post general anesthesia patient had no further symptoms of the corneal abrasion. However, the patient indicated that the first 48 hours post-delivery were extremely uncomfortable and that the discomfort from her eye exceeded that of the cesarean section wound.

## Discussion

The risk of exposure keratopathy in anesthetized patients, particularly those with incomplete eyelid closure, is significant [6] and is likely compounded by the presence of eyelash extensions. Exposure keratopathy, characterized by defects in the corneal surface epithelium, is a common postoperative complaint, with an incidence ranging from 0.9 to 3.3 per 1,000 general anesthetics [7]. Factors such as surgeries involving head and neck regions, lateral or prone positioning, and lagophthalmos significantly increase this risk [8-9]. Under general anesthesia, approximately 60% of patients experience incomplete eyelid closure, exposing the cornea to air and increasing the likelihood of dryness and damage from foreign bodies [5]. While standard protective measures, including specifically designed dressings and topical ointments, are typically employed, eyelash extensions (as in our case) can hinder the effectiveness of these dressings, leading to lagophthalmos and exposure keratopathy.

Eyelash extensions can also contribute to baseline ocular vulnerabilities such as blepharitis and dry eye disease, both of which have been linked to a higher risk of perioperative corneal abrasions [10]. These conditions reduce tear production, exacerbating corneal vulnerability during anesthesia [5]. In our case, the combination of lagophthalmos, blepharitis, and dry eyes resulted in a significant postoperative complication, highlighting the need for both a comprehensive risk assessment prior to surgery and a risk mitigation protocol in patients with eyelash extensions.

Such preoperative risk assessments should involve the assessment of Bell’s reflex. A reflex characterized by the involuntary upward gaze and extorsion of the eye when the eyelid is closed. This reflex serves as a protective mechanism for the cornea and is present in about 75% of the population [11]. Additionally, a formal evaluation of whether the patient completely closes their eyes when they shut them would also offer the anesthetist valuable information for stratifying the risk of exposure during surgery.

The mechanical erosion risk posed by gauze application without adequate lubrication must also be addressed. The rigidity of eyelash extensions increases the likelihood of friction-related injuries from gauze which was observed in our case. To prevent a reoccurrence the application of a protective, viscous, oil-based ointment over the ocular surface would assist in protecting the corneal surface during surgery as well as using a gentler material to cover the eyes.

Current literature highlights several effective strategies for minimizing risks associated with eyelash extensions in the perioperative setting. Byrne et al. emphasize the removal of eyelash extensions prior to surgery as a key preventive measure. If unable to remove, they also recommend using soft oval eye pads secured over the closed eyelids, supplemented by ocular lubricants during the procedure [10]. Similarly, Roth et al. advocate for removal prior to surgery, and if unable, to apply gentle eyelid closure with cotton pads and bio-occlusive dressings, along with regular face examinations to ensure ocular protection [12]. A notable quality improvement program by Vetter et al. illustrates the effectiveness of a standardized eye protection protocol that included aqueous-based lubrication and clear occlusive dressings [13]. This initiative successfully reduced intraoperative corneal injury rates, suggesting that a similar approach tailored to patients with eyelash extensions could be beneficial.

Taking these factors into account, we recommend improving eye protection protocols for patients with eyelash extensions. This includes educating patients about the potential ocular risks associated with eyelash extensions during anesthesia and advising their removal before surgery if feasible. If removal is not possible, we suggest conducting a thorough preoperative risk assessment to evaluate whether the patient has an intact Bell’s reflex and can fully close their eyes. Intraoperatively, a protective, viscous oil-based ointment should be applied to the ocular surface, followed by a clear occlusive dressing. Additionally, regular examinations of the patient’s face should be performed to ensure the eyes are adequately closed.

## Conclusions

In conclusion, a multifaceted strategy that incorporates preoperative eyelid function assessments, appropriate lubrication, and enhanced protective measures may reduce the risks of exposure keratopathy and related complications in patients with eyelash extensions undergoing anesthesia. Given the likely increased frequency of these cases, ongoing research and protocol refinement are essential to optimize patient outcomes.

## References

[1] Singh A, Singh S (2024). Singh A, Singh S: Lash Extension Market Size. Lash Extension Market - By Type (Mink, Human Hair, Silk, and Synthetic) By Length (Up to 5 mm, 5mm -10 mm, and more than 10.

[2] Masud M, Moshirfar M, Shah TJ, Gomez AT, Avila MR, Ronquillo YC (2019). Eyelid cosmetic enhancements and their associated ocular adverse effects. Med Hypothesis Discov Innov Ophthalmol.

[3] Pereira MV, Glória AL (2010). Lagophthalmos. Semin Ophthalmol.

[4] Putnam CM (2016). Diagnosis and management of blepharitis: An optometrist's perspective. Clin Optom (Auckl).

[5] White E, Crosse MM (1998). The aetiology and prevention of peri-operative corneal abrasions. Anaesthesia.

[6] Demirci H, Frueh BR (2009). Palpebral spring in the management of lagophthalmos and exposure keratopathy secondary to facial nerve palsy. Ophthalmic Plast Reconstr Surg.

[7] Roth S, Thisted RA, Erickson JP, Black S, Schreider BD (1996). Eye injuries after nonocular surgery. A study of 60,965 anesthetics from 1988 to 1992. Anesthesiology.

[8] Yu HD, Chou AH, Yang MW, Chang CJ (2010). An analysis of perioperative eye injuries after nonocular surgery. Acta Anaesthesiol Taiwan.

[9] Batra YK, Bali IM (1977). Corneal abrasions during general anesthesia. Anesth Analg.

[10] Byrne M. If Looks Could Kill (2024). If Looks Could Kill: Anesthetic Implications of Cosmetic Enhancements. https://www.apsf.org/article/if-looks-could-kill-anesthetic-implications-of-cosmetic-enhancements/.

[11] Roth S, Moss HE, Vajaranant TS, Sweitzer B (2022). Perioperative care of the patient with eye pathologies undergoing nonocular surgery. Anesthesiology.

[12] Vetter TR, Ali NMK, Boudreaux AM (2012). A case-control study of an intraoperative corneal abrasion prevention program: holding the gains made with a continuous quality improvement effort. Jt Comm J Qual Patient Saf.

[13] Neimkin MG, Holds JB (2016). Evaluation of eyelid function and aesthetics. Facial Plast Surg Clin North Am.

